# Perivascular adipose tissue inflammation in abdominal aortic aneurysm: An underrecognized contributor

**DOI:** 10.7555/JBR.39.20250415

**Published:** 2026-09-28

**Authors:** Jiayi Chen, Yang Xue, Liuhua Zhou, Liansheng Wang

**Affiliations:** Department of Cardiology, the First Affiliated Hospital of Nanjing Medical University, Nanjing, Jiangsu 210029, China

**Keywords:** abdominal aortic aneurysm, adipose tissue, inflammation, adipokine, perivascular adipose tissue

## Abstract

Abdominal aortic aneurysm (AAA) is a life-threatening vascular disorder characterized by localized dilation of the abdominal aorta and a high mortality rate once rupture occurs. At present, therapeutic approaches for AAA are primarily confined to operative repair. Thus, a comprehensive understanding of its pathogenesis is crucial for the development of novel pharmacological interventions. Although the underlying mechanisms remain incompletely defined, several critical pathological features have been identified, including inflammation, extracellular matrix degradation, and loss of vascular smooth muscle cells. In recent years, perivascular adipose tissue (PVAT), an active endocrine and paracrine organ surrounding blood vessels, has emerged as an essential regulator of various cardiovascular diseases. PVAT has been shown to remodel the extracellular matrix and influence vascular smooth muscle cell behavior by establishing a proinflammatory milieu, thereby affecting the structural integrity and reactivity of the arterial wall. This review summarizes recent research advances in PVAT-driven inflammation in AAA and highlights the translational potential for clinical applications, aiming to provide new insights for future research.

## Introduction

Abdominal aortic aneurysm (AAA) is generally defined as a permanent focal dilation of the abdominal aorta to ≥ 3.0 cm or ≥ 1.5 times the expected normal diameter^[1–2]^. Risk factors for AAA include older age, male sex, hypertension, smoking, coronary heart disease, family history, and obesity^[3–4]^. Patients with this progressive disease are often asymptomatic and may occasionally present with abdominal pain before rupture. Thus, AAA may easily be overlooked. Once rupture occurs, the mortality rate exceeds 65%^[5]^. Currently, the primary treatment options for AAA are surgical interventions, including open surgical repair and endovascular aneurysm repair (EVAR). Nevertheless, many AAAs are detected through imaging tests and remain below the threshold for surgical/endovascular intervention. Numerous studies have demonstrated that the risk of AAA rupture is not strictly proportional to its diameter, and even small AAAs may exhibit instability^[6–7]^. Moreover, patients with severe cardiopulmonary disease or coagulation dysfunction are often unsuitable candidates for surgical repair^[8]^. Therefore, there is an urgent need for safe and effective pharmacological therapeutic strategies to limit the progression of AAAs that are not indicated or unsuitable for surgical/endovascular intervention. A deeper understanding of the underlying mechanisms might help achieve this goal.

Recently, the potential role of perivascular adipose tissue (PVAT) in various cardiovascular diseases has attracted increasing attention. PVAT refers to the adipose tissue surrounding nearly all blood vessels^[9]^. It was once considered to serve merely as a mechanical support for blood vessels. However, exciting new evidence has accumulated highlighting the function of PVAT as a complex endocrine and paracrine organ capable of secreting inflammatory cytokines and vasoactive substances^[10]^, and its association with key pathological features of AAA^[11]^.

This review summarizes the research progress on PVAT in the pathogenesis and progression of AAA, focusing on its potential translational value in clinical practice.

## Characteristics of PVAT

Adipose tissue can be divided into three types according to function and appearance. White adipose tissue (WAT) is primarily located in subcutaneous and visceral sites, storing excess energy in the form of triglycerides. It can secrete adiponectin, leptin, interleukin (IL), tumor necrosis factor (TNF), and other cytokines that affect metabolism^[12]^. Brown adipose tissue (BAT) is mainly distributed in the interscapular region. It highly expresses uncoupling protein-1 (UCP-1) and is involved in maintaining body temperature, which is of great significance for infants and small mammals. The activation of UCP-1 facilitates inner mitochondrial membrane leakage, disrupts the coupling between electron transport and ATP production, and finally results in the proton motive force being released as thermal energy^[13]^. In addition to these two types, there is also a third adipose phenotype, beige adipose tissue (BeAT), which is derived from white fat under specific stimuli, such as cold exposure and agonists of β-adrenergic receptor or peroxisome proliferator-activated receptor γ (PPARγ)^[14]^. BeAT expresses UCP-1 and has the ability to generate heat as well, but shares the same precursor with WAT^[15]^.

The phenotype of PVAT depends on its specific anatomical location^[16]^. In rodent models and humans, thoracic PVAT is generally regarded as BAT-like^[17]^, whereas abdominal PVAT more closely resembles WAT^[18–20]^. One study generated mice lacking PVAT around the aorta and mesenteric arteries and discovered that they retained normal BAT in the interscapular region and WAT in the gonadal, inguinal, and subcutaneous areas. This raised the intriguing hypothesis that PVAT of the aorta might originate from a distinct lineage separate from both BAT and WAT, and might represent a "fourth type" of adipose tissue^[10]^. While this concept remains controversial, most evidence so far is derived from mouse models, and direct validation in humans is still lacking. Lineage tracing is the gold standard for determining developmental origin, but such an approach is technically and ethically infeasible in human studies. Consequently, current human research mainly relies on comparing transcriptomic, proteomic, and phenotypic evidence to infer similarities or differences between PVAT and other adipose depots; however, it cannot directly prove their developmental origin.

PVAT is in direct contact with the adventitial layer of the vessel wall and lacks a clear boundary with it, which forms the basis for its effect on vascular homeostasis^[21]^. It is primarily composed of mature adipocytes and preadipocytes, various immune cells, mesenchymal stem cells, and collagen fibers^[22]^. Under physiological conditions, beyond its structural support, PVAT regulates vascular tone and endothelial function by releasing mediators such as nitric oxide (NO), adipokines, and angiotensin peptides; it also influences vascular smooth muscle cell (VSMC) proliferation and migration. Under pathological conditions, such as obesity or hypertension, this regulatory capacity is impaired. Both dysregulated adipokine secretion and oxidative stress activation weaken PVAT's anticontractile effect, ultimately contributing to vascular injury^[12–23]^. The response of PVAT to pathological stimuli is also sex-specific. Though research on the role of androgens in PVAT is limited, the loss of estrogen after menopause may exacerbate PVAT dysfunction and worsen aortic outcomes. Furthermore, male abdominal PVAT appears to contain more immune cells, which may explain the significant male-to-female disparity observed in the incidence of AAA^[24]^. Aging is also closely associated with PVAT, with increased oxidative stress and inflammation observed in obese aged mice^[25]^. Recent studies have indicated changes in PVAT function or morphology in aging and various vascular diseases. Still, it remains unclear whether these changes are the cause or consequence of the disease process^[26]^.

## PVAT inflammation and AAA

PVAT inflammation refers to the enhanced infiltration of inflammatory cells and the upregulated secretion of proinflammatory cytokines and adipokines under pathological conditions, thereby leading to PVAT dysfunction^[27–28]^. This inflammatory status has been shown to be pathogenic in many vascular diseases, including hypertension and atherosclerosis^[29]^. Accumulating preclinical and clinical evidence indicates that PVAT inflammation may be associated with the development and progression of AAA. For instance, an increase in plasma adiponectin levels can inhibit inflammation in PVAT and the aorta in mice, and reduce the incidence of AAA and its progression to advanced stages^[30]^. In humans, imaging data from the Framingham Heart Study demonstrated that increased PVAT volume was correlated with larger aortic dimensions even in individuals without cardiovascular disease, suggesting that PVAT expansion may participate in the subclinical stages of aortic remodeling^[31]^. Furthermore, transcriptomic analyses revealed that PVAT surrounding the dilated segment in AAA patients exhibited a distinct gene expression profile compared with non-dilated regions, with the difference becoming more significant as the aneurysm diameter increased. Cell-type enrichment analysis further indicated that both resting and activated mast cells were associated with dilated PVAT (D-PVAT) in the small AAA subgroup (maximum diameter 45.6–49.5 mm), while activated dendritic cells were associated with D-PVAT in the large subgroup (maximum diameter 65–74.6 mm)^[32]^. Histological studies investigating human PVAT and the necrotic adipose tissue adjacent to AAA found substantial accumulation of neutrophils, macrophages, mast cells, and T cells^[33]^. Studies of human samples obtained during AAA repair surgery showed that CD45^+^ leukocyte counts in PVAT were positively correlated with those in the arterial wall^[34]^, suggesting coordinated inflammatory cell accumulation in PVAT and the adjacent arterial wall and supporting a potential contribution of outside-in inflammation. In previous studies, considerable attention has been focused on the "inside-out" inflammation, with the notion that inflammatory cell infiltration is a process that originates from the luminal side of the vascular wall and radiates outwards. However, subsequent evidence has emphasized the "outside-in" inflammation in AAA. Although the density of vasa vasorum is relatively low in the human abdominal aorta, neovascularization frequently occurs within the adventitia and outer media of aneurysmal aortas, accompanied by inflammatory cell infiltration^[35]^. The above evidence suggests that PVAT, which lies in proximity to the aortic adventitia and is metabolically active and highly vascularized, may participate in amplifying this outside-in inflammatory response.

## Formation and development of PVAT inflammation in AAA

During the pathological process of AAA, inflammatory cells and adipocytes in PVAT collectively contribute to the formation of an inflammatory microenvironment (***Fig. 1***).

**Figure 1:**
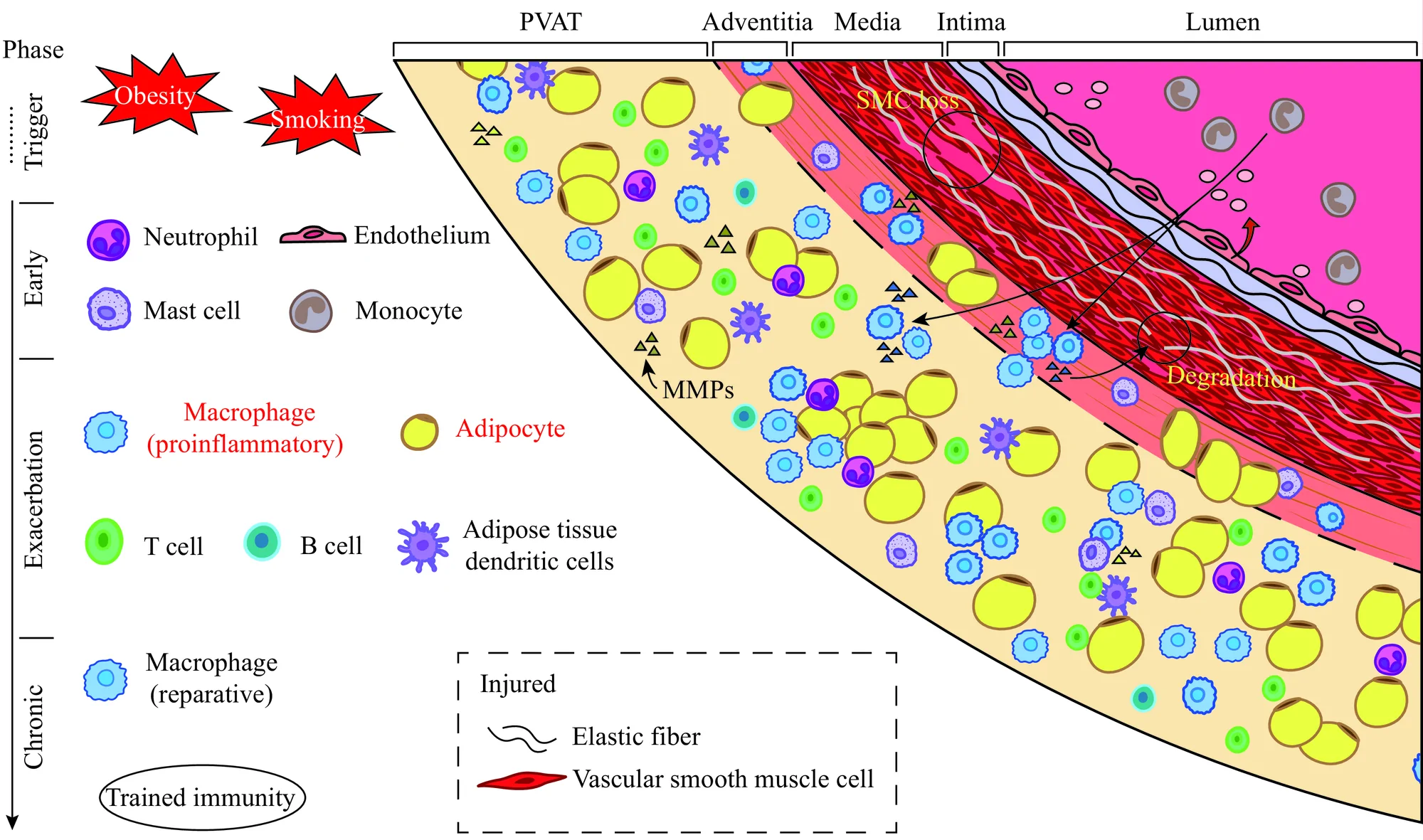
Distribution of PVAT inflammatory cells and main actors at each stage. Obesity and smoking are triggers for PVAT inflammation. Neutrophils may participate in early inflammation. Endothelial cells and mast cells can both promote the movement of monocytes from the circulation into PVAT. Proinflammatory macrophages can interact with adipocytes through inflammatory factors and adipokines, contributing to continuous aggravation of inflammation. T/B lymphocytes and adipose tissue dendritic cells also assist in this process. During later phases, macrophage phenotypes may shift toward reparative states, while trained-immunity-associated epigenetic changes may contribute to persistent inflammatory responses. Excessive MMPs often lead to the degradation of elastic fibers, and the inflammatory microenvironment can also cause the loss of smooth muscle cells. Abbreviations: MMP, matrix metalloproteinase; PVAT, perivascular adipose tissue; SMC, smooth muscle cell.

Neutrophils are generally considered early markers of acute inflammation and are recruited to the injured tissue before monocytes^[36]^. Wang *et al*^[37]^ analyzed PVAT samples collected from regions adjacent to AAA lesions and from non-dilated aortic segments in 30 patients, and found that differentially expressed genes were mainly enriched in pathways related to neutrophil chemotaxis and IL-17 signaling. Similarly, Folkesson *et al*^[33]^ reported excessive infiltration of neutrophils and mast cells in PVAT obtained from AAA patients. However, whether neutrophils act as the actual initiators of PVAT inflammation in AAA remains uncertain. Considering the strong association between AAA and obesity, as well as the frequent use of high-fat diet (HFD) in AAA animal models, evidence from obesity-related studies may provide valuable insights. In obese mice, transient neutrophil infiltration into adipose tissue occurs as early as three days after HFD initiation. They are attracted by IL-8, and can interact directly with adipocytes by forming complexes between CD11b and intercellular adhesion molecule-1 (ICAM-1)^[38]^. It has been reported that neutrophils also secrete some proteases, notably neutrophil elastase (NE), which are involved in propagating inflammatory signaling. Talukdar and colleagues demonstrated that NE activated Toll-like receptor 4 (TLR4), thereby promoting subsequent macrophage recruitment and M1 macrophage polarization, which in turn amplified the release of proinflammatory cytokines from both macrophages and adipocytes^[39]^. Whether a similar neutrophil-driven inflammatory cascade operates in PVAT inflammation during AAA development requires further investigation.

Mast cells (MCs) are also early-responding cells in PVAT inflammation. As components of the innate immune system, they can release proinflammatory mediators such as TNF-α, IL-4, IL-6, and C-C motif chemokine ligand 2 (CCL2) through degranulation, thereby amplifying local inflammatory responses. In AAA patients, MCs are enriched not only within the adventitia and media, but also in the surrounding PVAT, where their abundance is positively associated with the diameter of aneurysms^[40]^. Recent transcriptomic and bioinformatics studies further suggest that PVAT from AAA patients exhibits higher expression of mast cell-related genes. Guo *et al*^[41]^ analyzed clinical datasets and reported that the early growth response 1 (EGR1) was upregulated in PVAT, which was further validated in an AAA mouse model. Correlation analysis suggested that EGR1 expression was positively correlated with activated mast cell infiltration. Similarly, Ding *et al*^[42]^ identified the FOS proto-oncogene as a molecular marker distinguishing dilated from non-dilated PVAT in AAA patients, with positive correlations with MC activation as well as with the recruitment of monocytes and follicular helper T cells (Tfh). These findings imply a potential involvement of mast cells in orchestrating PVAT-derived inflammation during AAA development, providing preliminary molecular evidence that warrants further mechanistic investigation.

Macrophages are central effectors in PVAT inflammation. They are traditionally classified into proinflammatory M1 and anti-inflammatory M2 subsets. Although the M1/M2 framework is conceptually useful, macrophage phenotypes *in vivo* exist along a continuum and are highly context-dependent. Crucially, macrophage infiltration and polarization in AAA are dynamic rather than static. In a murine AAA model, the suprarenal aortas maintained a high M1/M2 ratio until 28 days after the infusion of angiotensin Ⅱ (Ang Ⅱ)^[43]^. However, when the infusion was extended to 56 days, immunostaining showed more M2 markers than M1 in the vascular wall^[44]^, suggesting a temporal shift toward a more reparative macrophage phenotype at later stages. Within PVAT, macrophage recruitment is driven in large part by CCL2, a principal chemokine for monocytes. Police *et al*^[4]^ reported that genetic obesity or a high-fat diet combined with Ang Ⅱ increased AAA incidence in mice, which was correlated with higher CCL2 release from PVAT and greater macrophage infiltration. Meanwhile, adipokines can amplify this axis. Fat-specific protein 27 (FSP27), a lipid droplet-associated protein in rodents that promotes adipose expansion, has been shown to increase CCL2 expression *via* activation of c-Jun N-terminal kinase (JNK) signaling in adipocytes, thereby upregulating the proportion of macrophages migrating *via* the CCL2/C-C motif chemokine receptor 2 (CCR2) axis^[45]^.

Serum amyloid A (SAA) is the precursor of amyloid A, the deposition of which is involved in low-grade inflammation associated with chronic diseases such as Alzheimer's disease and atherosclerosis. In patients with severe leptin-receptor-deficiency-related obesity, adipocytes directly increase circulating SAA levels and even become the primary source of SAA^[46]^. Webb *et al*^[47]^ first confirmed that endogenous SAA deficiency reduced Ang Ⅱ-induced AAA in *Apoe*^*−/−*^ mice. Additionally, overexpression of SAA in PVAT enhanced macrophage infiltration in the adjacent aorta^[48]^. Leptin is encoded by the obesity gene (*ob*), and its circulating concentrations generally reflect adipose mass^[49]^. Evidence from murine AAA models suggests that the secretion of leptin and fatty acid-binding protein 4 (FABP4) by adipocytes in PVAT can upregulate IL-18, the canonical IL-18 receptor (IL-18R), and Na-Cl co-transporter (NCC), a newly identified IL-18 receptor in AAA lesions. Binding of IL-18 to these two receptors promotes IL-18 signaling in macrophages and T cells, thereby enhancing the expression of inflammatory mediators, including IL-6, interferon-γ (IFN-γ), and TNF-α^[50]^.

Both T lymphocytes and B lymphocytes have been detected in AAA tissues, but T cells play a dominant role in the pathogenesis^[34]^. According to the CD molecules expressed on their surface, T cells can be categorized into CD4^+^ and CD8^+^ subsets, and the former constitutes the majority of the infiltrating T cells. T helper 17 (Th17) cells, a proinflammatory subset of the CD4^+^ T cells, are increased in adipose tissue of mice under HFD conditions. Chen *et al*^[51]^ revealed that immature adipose tissue dendritic cells (ATDCs) released higher levels of IL-6, transforming growth factor-β (TGF-β), and IL-23 in mice, promoting the differentiation of CD4^+^ T cells into Th17 cells. The T cell receptor (TCR) is composed of α and β chains, and the latter is crucial for antigen recognition and immune response. Piacentini *et al*^[52]^ revealed that the TCRβ chain repertoire in human PVAT differed between large and small AAAs, and speculated that the initiation of T cells might be in response to multiple autoantigens produced by the degradation of extracellular matrix (ECM) and cell death. Smoking is a major clinical risk factor for human AAA that is not inherently reproduced in most commonly used AAA animal models. Ceramide is a bioactive lipid molecule that can be elevated by smoking and has the ability to promote sterile inflammation, possibly through signaling pathways involving proinflammatory cytokines such as TNF-α, IL-1, and IL-6^[53]^. In human AAA, the concentration of ceramide in PVAT is positively correlated with the T cell marker CD3 in the adventitia, but negatively correlated with neutrophils, mast cells, and macrophages, suggesting that ceramide may be particularly associated with T-cell recruitment in this setting^[33]^. Although the precise mechanism remains unclear, previous research has shown that the cell-permeable C6-ceramide can enhance Th1 differentiation and activate IFN-γ transcription *via* the cyclooxygenase-2 (COX-2)/T-bet signaling axis *in vitro*^[54]^. Interestingly, Sagan *et al*^[55]^ also reported that the current smoking status of AAA patients was significantly associated with the number of CD3^+^ T cells within the intraluminal thrombus (ILT). Given that inflammation in AAA is not localized but rather represents a complex and dynamic process, communication may exist between intraluminal and extravascular inflammatory sources. ILT may therefore influence PVAT inflammation, or vice versa. Whether ceramides play a role in this potential crosstalk is an intriguing question that deserves further investigation.

In addition to the classical infiltration of lymphocytes and myeloid cells, recent studies have proposed the presence of "trained immunity" in PVAT. This refers to the long-term functional reprogramming of innate immune cells caused by exogenous or endogenous stimuli, which then exhibit effects similar to the memory-like features of adaptive immunity^[56]^. Piacentini *et al*^[57]^ predicted 29 histone modification markers associated with large AAAs and 18 markers associated with small AAAs in humans. In particular, in the D-PVAT of large AAAs, many histone modification markers are characteristic of trained immunity, including H3K27ac/H3K4me3/H3K4me1. These epigenetic modifications may be involved in maintaining the chronic inflammatory environment observed in PVAT.

## Mechanisms of PVAT inflammation promoting AAA

Inflammation has been confirmed to contribute to the occurrence of the majority of AAAs^[58]^. Although many studies have demonstrated the association between PVAT inflammation and AAA, the specific mechanisms have not yet been thoroughly studied. However, it has been found that PVAT inflammation can affect AAA in at least two major aspects: promoting ECM degradation and inducing VSMC abnormalities, thus damaging the aortic media and adventitia.

### PVAT inflammation promotes ECM degradation

The aortic wall is primarily composed of structural elements, including collagen, elastin, proteoglycans, and matrix glycoproteins^[59]^; its homeostasis relies on the dynamic balance between proteases like matrix metalloproteinases (MMPs) and their endogenous inhibitors, tissue inhibitors of metalloproteinases (TIMPs). MMPs are a family of zinc-dependent proteases. Among them, MMP-1, MMP-2, MMP-3, MMP-9, and MMP-12 are believed to be closely associated with AAA^[60]^; their excessive activation accelerates the degradation of elastin and collagen, weakening the aortic wall and promoting vascular dilation.

Inflammatory cells, especially macrophages, are major sources of these proteases. Macrophages recruited through the CCL2/CCR2 axis predominantly secrete MMP-12^[45]^, which degrades collagen Ⅻ, fibronectin, periostin, and other substrates. In *Apoe^−/−^* mice, selective overexpression of endothelin-1 (ET-1) in endothelial cells facilitated the recruitment of circulating monocytes to the vessel wall and PVAT, where they differentiated into macrophages and contributed to increased MMP-2 expression^[61]^. Sakaue *et al*^[62]^ revealed that activation of the angiotensin Ⅱ type 1a receptor (AT1a) in PVAT stimulated osteopontin (OPN) expression in adipocytes, which in turn facilitated macrophage infiltration and enhanced MMP-2 and MMP-9 activity in AAA mice. Transplantation of PVAT lacking either AT1a or OPN significantly reduced vascular inflammation and aneurysm formation^[62]^. Tazume *et al*^[63]^ discovered that angiopoietin-like protein 2 (ANGPTL2) from infiltrating macrophages could activate the nuclear factor kappa-B (NF-κB) cascade by promoting inhibitor of kappa B degradation, and upregulate MMP-9 expression in the CaCl_2_-induced mouse AAA model. Moreover, adventitial mast cells enhanced macrophage-derived MMP-9 production in AAA patients and rodents. Further *in vitro* studies showed that this was achieved either through direct contact or *via* IFN-γ signaling^[64]^.

Besides proteases, Zhang *et al*^[65]^ employed gene expression microarray analysis and uncovered elevated levels of platelet-derived growth factor-D (PDGF-D) in PVAT of *ob/ob* mice. PDGF-D was predominantly expressed by adipocytes and spurred adventitial fibroblast activation through the TGF-β/Smad signaling pathway. It exacerbated vascular inflammation by enhancing the infiltration of CD68^+^ macrophages and then drove adventitial reconfiguration^[65]^.

### PVAT inflammation modulates VSMCs

Inflammatory responses in PVAT also profoundly influence the fate of VSMCs in AAA. Under physiological conditions, VSMCs maintain a contractile phenotype to preserve aortic wall integrity. When stimulated by inflammatory factors, VSMCs undergo phenotypic switching toward a synthetic state, characterized by the downregulation of contractile markers together with enhanced proliferative and migratory capacities^[66]^. This synthetic phenotype weakens normal contractile function and generates more MMPs. Li *et al*^[67]^ reported that hypertrophic adipocytes in inflammatory PVAT also secreted extracellular vesicles (EVs) enriched in microRNAs, which were internalized by VSMCs in HFD-induced obese mice. miR-221-3p inhibits peroxisome proliferator-activated receptor γ coactivator 1α (PGC-1α), an essential regulatory factor of mitochondrial function in vascular diseases, at the transcriptional and post-transcriptional levels, thereby ultimately leading to reduced VSMC contractile gene expression and promoting VSMC proliferation and migration^[67]^.

Leptin is another factor released by adipose tissue under inflammatory conditions. There is evidence suggesting that in rats with metabolic syndrome, leptin binds to the upregulated aortic leptin receptor and activates the p38 mitogen-activated protein kinase (MAPK) signaling pathway, thereby downregulating the differentiation markers (α-smooth muscle actin, calponin, and smooth muscle myosin heavy chain), and upregulating the proliferating cell nuclear antigen (PCNA) in VSMCs^[68]^. In addition, visfatin secreted by PVAT can also regulate the VSMC phenotype through the MAPK pathway. It is highly expressed in the aortic PVAT of Sprague-Dawley rats and monkeys compared with other adipose tissues. Visfatin possesses the enzymatic activity of nicotinamide phosphoribosyltransferase (NAMPT), which can utilize nicotinamide to synthesize nicotinamide mononucleotide (NMN), finally activating extracellular signal-regulated kinase 1/2 (ERK1/2) and p38 MAPK signaling and driving the proliferation of human aortic VSMCs *in vitro*^[69]^.

Progressive VSMC loss is another pathological feature of AAA^[70]^. This quantitative loss is usually caused by the apoptosis of VSMCs and finally leads to thinning of the media. Reactive oxygen species (ROS) are often inducers of apoptosis^[71]^. IL-6 has been reported to upregulate Ang Ⅱ type 1 (AT1) receptors, thereby enhancing the effect of angiotensin in C57 mice and increasing ROS production and oxidative stress in VSMCs^[72]^. The reduction of anti-inflammatory adipokines in obese PVAT, such as adiponectin, can lead to an increase in the production of ROS, such as O2^•−^ and H_2_O_2_^[73]^. Huang *et al*^[74]^ found that compared with PVAT surrounding the thoracic aorta, abdominal PVAT of AAA mice lacked the anti-apoptotic protein cartilage oligomeric matrix protein (COMP), which could significantly increase survivin protein levels in VSMCs and reduce VSMC apoptosis. From another perspective, VSMC death can also promote inflammation in turn. Although apoptosis has traditionally been regarded as non-inflammatory, Yamanouchi *et al*^[75]^ demonstrated that CCL2 mediated the chemoattractant effect of apoptotic VSMCs and triggered macrophage infiltration during mouse aortic SMC death *in vitro*.

From a biomechanical perspective, the role of PVAT inflammation in AAA development appears to follow a progressive process. Local mechanical stress itself can act as a proinflammatory stimulus, enhancing matrix degradation and inflammatory cell recruitment within the aortic wall^[76]^. Extending this concept, elevated wall tension and heterogeneous strain distribution may induce adipocyte hypoxia, oxidative stress, and cytokine release in the adjacent PVAT. Once established, PVAT inflammation further aggravates the local biomechanical environment of the aneurysmal wall. It promotes elastin and collagen degradation by activating MMPs and disrupts ECM organization. Meanwhile, it drives the phenotypic switching and apoptosis of VSMCs, ultimately weakening the mechanical strength of the tunica media (***Fig. 2***). The resulting structural deterioration increases local wall tension, forming a vicious cycle in which PVAT inflammation and wall stress mutually reinforce each other. This mechanical–inflammatory feedback loop accelerates aneurysm expansion and compromises vascular stability.

**Figure 2:**
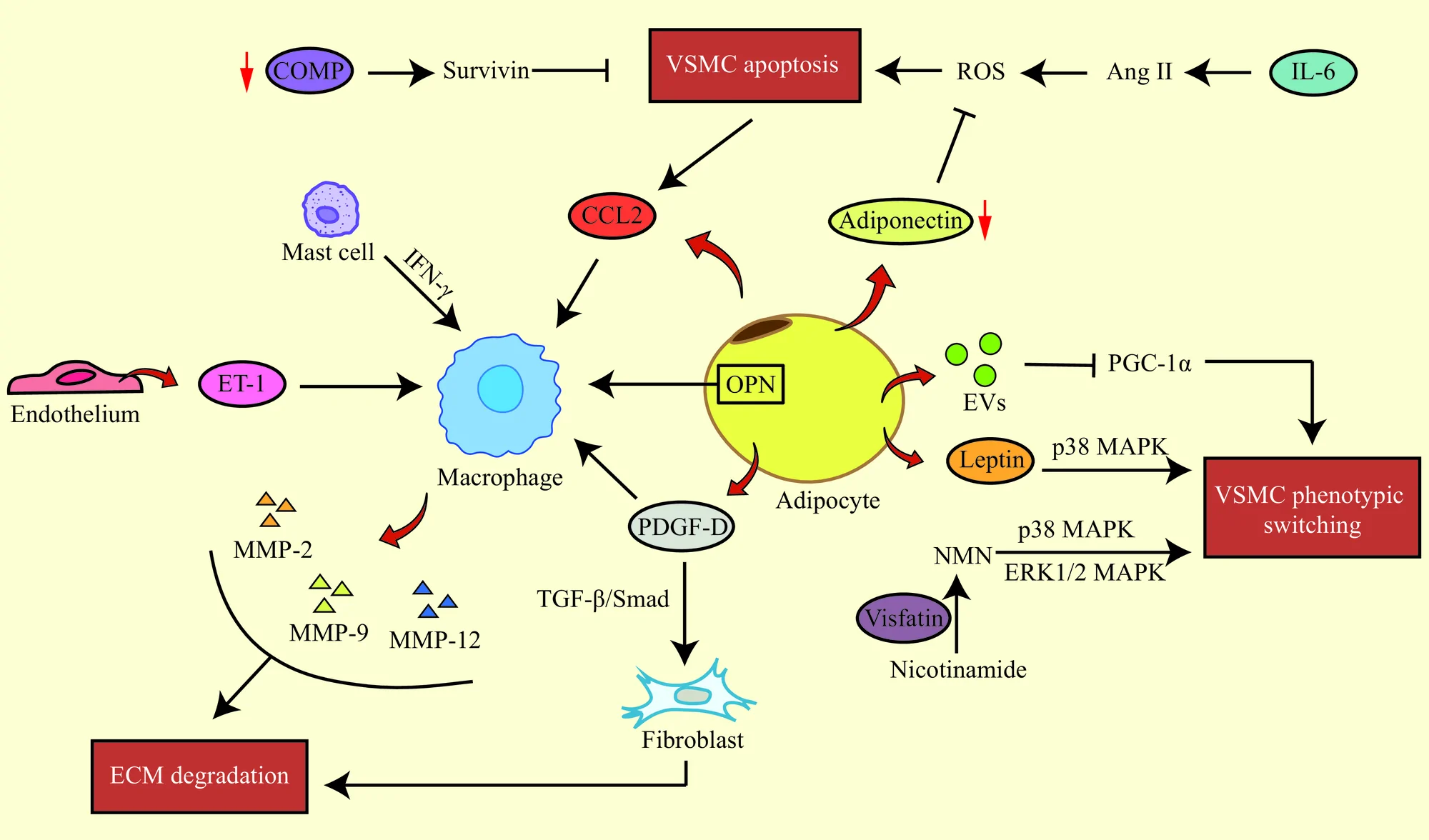
Mechanisms of PVAT inflammation promoting AAA. Macrophage-derived MMPs contribute importantly to ECM degradation: endothelial cells secrete ET-1 to promote macrophage recruitment and increase MMP-2. Mast cells increase MMP-9 through IFN-γ signaling. CCL2 can be secreted by adipocytes, and macrophages derived from this mainly secrete MMP-12. Overexpression of OPN in adipocytes enhances MMP-9 and MMP-12. PDGF-D secreted by adipocytes increases macrophage infiltration and stimulates fibroblast activation through TGF-β/Smad. EVs secreted by adipocytes promote the proliferation and migration of vascular smooth muscle cells. Both visfatin and adipocyte-derived leptin promote phenotypic transformation of VSMCs through the MAPK pathway. IL-6 upregulates AT1 receptor expression, thereby enhancing Ang Ⅱ signaling and ROS production, which ultimately promotes VSMC apoptosis. A decrease in adiponectin can also increase ROS. In the cited AAA mouse model, abdominal PVAT showed reduced COMP compared with thoracic PVAT; COMP may protect VSMCs from apoptosis by increasing survivin. Apoptosis of VSMCs can promote inflammation in turn through CCL2. Abbreviations: Ang Ⅱ, angiotensin Ⅱ; CCL2, C-C motif chemokine ligand 2; COMP, cartilage oligomeric matrix protein; ECM, extracellular matrix; ERK, extracellular signal-regulated kinase; ET-1, endothelin-1; EV, extracellular vesicle; MAPK, mitogen-activated protein kinase; MMP, matrix metalloproteinase; NMN, nicotinamide mononucleotide; OPN, osteopontin; PDGF-D, platelet-derived growth factor-D; PGC-1α, peroxisome proliferator-activated receptor γ coactivator 1α; PVAT, perivascular adipose tissue; ROS, reactive oxygen species; TGF-β, transforming growth factor-β; VSMC, vascular smooth muscle cell.

## Translation of PVAT inflammation studies

### Imaging assessment of PVAT inflammation in human AAA

Some researchers have proposed that changes in PVAT density or attenuation on computed tomography (CT) may reflect its inflammatory state and provide insights into AAA progression and prognosis. In this context, CT-derived "density" generally refers to attenuation characteristics measured in Hounsfield units (HU), although terminology varies across studies. For instance, Dias-Neto *et al*^[77]^ conducted a multicenter retrospective case-control study to examine differences in PVAT density within a 10-mm circumferential region surrounding the aorta. The study included individuals with untreated asymptomatic AAA or aortoiliac occlusive disease, and those without aortic pathology. The results indicated that patients with asymptomatic AAA exhibited greater variability in PVAT density, with significantly higher PVAT density surrounding the aneurysm sac compared with the morphologically normal neck region. The authors hypothesized that the PVAT density might reflect both a higher adipocyte content and a greater inflammatory burden^[77]^. Similarly, Ginzburg *et al*^[78]^ examined changes in PVAT density adjacent to the aorta, uniquely incorporating artificial intelligence to assist in distinguishing the aorta from surrounding adipose tissue. They found that changes in PVAT density were evident both between AAA patients and controls, and between aneurysmal and non-aneurysmal segments within AAA patients^[78]^. In a study by Zhang *et al*^[79]^, which included AAA patients with or without ILT, multivariable Cox regression analysis demonstrated that increased PVAT attenuation was independently associated with AAA progression, including in patients with ILT. Debono *et al*^[80]^ evaluated PVAT attenuation in patients with asymptomatic or symptomatic AAAs, as well as in controls without aneurysms. Their findings, however, were inconsistent with those of previous studies. They reported that PVAT attenuation did not differ significantly between asymptomatic AAA patients and controls, whereas symptomatic AAA patients (with or without rupture) exhibited markedly higher attenuation compared with both asymptomatic patients and controls^[80]^.

Among the four studies summarized in ***Table 1***, the central conflict lies in the conclusions regarding asymptomatic AAA patients. Several factors may account for this discrepancy. First, the two studies exhibit notable cohort heterogeneity. Debono *et al*^[80]^ conducted a single-center study with a smaller sample size and enrolled patients with smaller AAA diameters than those in the study of Dias-Neto *et al*^[77]^, potentially reflecting a lower inflammatory state. Second, their CT measurement strategies also differed substantially: Dias-Neto *et al*^[77]^ used a 10-mm region of interest from the aortic wall, which may have included non-adipose structures, whereas Debono *et al*^[80]^ applied a validated semi-automated method restricted to a 2-mm periaortic zone. Furthermore, given that body mass index (BMI) and systemic adipose inflammation may influence PVAT attenuation, the lack of explicit adjustment for BMI or statin/anti-inflammatory medication use in Debono's multivariable model may have contributed to differences in the observed associations. Together, these factors highlight the need for standardized CT acquisition and analysis protocols, as well as consistent adjustment for clinical confounders, to enhance the reliability and interpretability of PVAT attenuation as a marker of inflammatory activity in AAA.

**Table 1:** Summary and comparison of studies investigating the relationship between PVAT density changes and AAA progression

Years	Subjects	Center	Conclusions	References
2009–2016	Non-treated asymptomatic AAA (*n* = 140), aortoiliac occlusive disease (*n* = 104), and individuals without aortic pathology (*n* = 97)	Multicenter	AAA patients presented higher intra-individual PVAT differences.	[77]
2016–2020	AAA (*n* = 100) and control (without underlying aortic disease; *n* = 100)	Single-center	Alterations in PVAT density were observed both between AAA patients and controls and between aneurysmal and non-aneurysmal segments in AAA patients.	[78]
2015–2022	AAA with or without ILT (*n* = 167)	Multicenter	Elevated PVAT attenuation was independently associated with AAA progression, including in AAA cases with ILT.	[79]
2018–2022	Asymptomatic AAA (*n* = 40), symptomatic AAA (*n* = 30), and control with normal abdominal aorta (*n* = 18)	Single-center	PVAT attenuation was not increased in stable AAAs, but was increased in symptomatic AAA patients.	[80]
Abbreviations: AAA, abdominal aortic aneurysm; ILT, intraluminal thrombus; PVAT, perivascular adipose tissue.				

### Pharmacological interventions targeting PVAT inflammation in AAA animals

Beyond prognostic prediction, therapeutic strategies targeting PVAT inflammation are also under active investigation. At present, no clinical pharmacological trials have specifically targeted PVAT inflammation in AAA. However, several agents have been shown in animal studies to attenuate aneurysm progression by modulating PVAT inflammation. Bao *et al*^[81]^ identified calbindin 2 (CALB2) as a plasma protein closely associated with AAA risk using multiple genetic approaches. CALB2 may participate in the regulation of TNF and chemokines within PVAT. Genistein, identified as a candidate compound associated with CALB2-related regulation, has been shown to reduce AAA incidence in mice and attenuate aortic wall injury^[81]^. Zhang *et al*^[82]^ investigated the effect of mirabegron (a β3-adrenergic receptor agonist) on both aortic aneurysm and aortic dissection. In the Ang Ⅱ-infused *Apoe^−/−^* mouse model of aortic aneurysm, mirabegron treatment significantly reduced the incidence and severity of aneurysm formation, as evidenced by decreased aortic dilation, reduced elastin fragmentation, and attenuated macrophage infiltration within the aortic wall. Mechanistically, mirabegron upregulated vascular endothelial growth factor C (VEGF-C) expression in PVAT, promoted local lymphangiogenesis, and enhanced drainage of PVAT inflammatory cells, including CD4^+^ T cells, CD8^+^ T cells, and CD11c^+^ cells, but this has not yet been demonstrated in human AAA^[82]^. Kunath *et al*^[83]^ demonstrated that metformin reduced Ang Ⅱ-induced aneurysm formation in non-diabetic mice, an effect that may be attributed to the inhibition of *Spp1* (encoding osteopontin) and *Mmp12* mRNA expression in PVAT. The selective mineralocorticoid receptor antagonist eplerenone effectively reduced the expression of TNF-α, IL-6, and MMP-2, as well as macrophage infiltration in PVAT of AAA mice, while improving the integrity and elasticity of the aortic wall^[84]^. Quinoline-Val-Asp-difluorophenoxymethylketone (Q-VD-OPh), a broad-spectrum caspase inhibitor, has been shown to inhibit caspase-dependent smooth muscle cell apoptosis and CCL2 production, thereby reducing subsequent macrophage infiltration and inflammation in AAA mice^[75]^.

### Pharmacological modulation of PVAT inflammation in other diseases

In addition, several agents, although not directly studied in AAA, have been reported to modulate PVAT inflammation. For instance, a clinical trial demonstrated that the PPARγ agonist pioglitazone reduced adipose tissue inflammation in insulin-resistant subjects by reducing mast cells and macrophages and improving vascularization^[85]^. Moreover, pioglitazone has been shown to directly induce apoptosis of macrophages in the subcutaneous adipose tissue of subjects with impaired glucose tolerance^[86]^. Specialized proresolving mediators (SPMs), such as resolvin D2 (RvD2), are increasingly recognized for their roles in controlling excessive inflammation. Rodrigues-Diez *et al*^[87]^ reported elevated expression of the RvD2 receptor, G protein-coupled receptor 18 (GPR18), in aneurysm wall samples from human AAA patients. In an obese hypertensive mouse model, administration of RvD2 substantially reduced IL-6 expression and promoted M2 macrophage polarization in PVAT^[87]^. These findings underscore the therapeutic potential of not only suppressing proinflammatory signaling but also promoting proresolving pathways. ODN2088, a pharmacological inhibitor of Toll-like receptor 9 (TLR9), has been shown to attenuate NF-κB signaling, restore the anticontractile function of PVAT, reduce inflammation and oxidative stress, and improve vascular tone in spontaneously hypertensive rats^[88]^. Similarly, propagermanium, an immunomodulatory agent with anti-inflammatory properties, has been demonstrated in diabetic rat models to restore PVAT anticontractile function by reducing inflammation and oxidative stress^[89]^.

Despite encouraging preclinical evidence, clinical data on PVAT-targeted therapies for AAA remain remarkably scarce (***Table 2***). Several factors may hinder this bench-to-bedside translation. First, although many agents have been shown to reduce AAA formation and rupture in rodent models, the underlying mechanisms have not been fully elucidated. Conducting clinical trials without a clear mechanistic foundation may entail potential risks. Second, the systemic adipose network is highly interconnected, and achieving selective modulation of PVAT without off-target effects in other depots remains challenging. Nevertheless, the anatomical heterogeneity of PVAT might offer opportunities to overcome this barrier by allowing region-specific targeting approaches. Additionally, the optimal dosage and administration strategies for PVAT-targeted drugs during translation from animal studies to humans remain undefined. Future efforts should focus on conducting carefully designed early-phase clinical studies, including pharmacodynamic and dose-finding studies, to delineate the therapeutic and safety windows of candidate agents, while simultaneously developing precise delivery systems that minimize systemic exposure.

**Table 2:** Pharmacological intervention for PVAT inflammation and the challenges of translation

Interventions	Primary mechanisms	Models	Challenges
Genistein	Participate in the regulation of TNF and chemokines	AAA mouse (Ang Ⅱ + *Apoe^−/−^*)	The mechanism has not been fully elucidated; there are no mature, specific drugs for the CALB2 target.
Mirabegron	Promote PVAT lymphangiogenesis *via* adipocyte-derived VEGF-C	AA/AD mouse (*Apoe^−/−^* + Ang Ⅱ/BAPN + Ang Ⅱ)	Mirabegron is reported to induce adipocyte browning and may affect body weight in humans.
Metformin	Inhibit *Spp1* (encoding osteopontin) and *Mmp12* mRNA expression	AAA mouse (Ang Ⅱ + *Apoe^−/−^*)	Long-term rupture outcomes were not evaluated; the dose used in non-diabetic human patients is unknown.
Eplerenone	Reduce the expression of TNF-α, IL-6, and MMP-2, as well as macrophage infiltration	AAA mouse (Ang Ⅱ + BAPN)	PVAT-specific delivery was not addressed; systemic mineralocorticoid blockade may cause electrolyte disturbances and off-target effects.
Q-VD-OPh	Inhibit caspase-dependent SMC apoptosis and downregulate CCL2, reducing subsequent macrophage infiltration	AAA mouse (Ang Ⅱ + *Apoe^−/−^*)	Systemic caspase inhibition lacks PVAT specificity and may cause off-target effects or toxicity.
Pioglitazone	Reduce mast cells and macrophages and improve vascularization	Clinical trial (insulin-resistant subjects)	The study had a small sample size and limited generalizability; systemic metabolic effects may confound local adipose changes.
Resolvin D2	Downregulate vascular expression of proinflammatory mediators (IL-6, TNF-α, CCL2)	Obese hypertensive mouse (Ang Ⅱ + HFD)	The model is not specific to AAA, limiting direct translational interpretation.
ODN2088	Attenuate NF-κB signaling and restore the anticontractile function of PVAT	Spontaneously hypertensive rat	Translation in AAA remains uncertain; TLR9 modulation may have unintended immune effects.
Propagermanium	Modulate the CCL2/CCR2-monocyte/ macrophage pathway and reduce CD36 expression in PVAT	Non-obese type 2 diabetic Goto-Kakizaki rat	Translation in AAA remains uncertain; PVAT measurements are functional or phenotypic but lack detailed molecular profiling.
Abbreviations: AAA, abdominal aortic aneurysm; AA/AD, aortic aneurysm and dissection; Ang Ⅱ, angiotensin Ⅱ; Apoe, *apolipoprotein E*; BAPN, β-aminopropionitrile; CALB2, calbindin 2; CCL2, C-C motif chemokine ligand 2; CCR2, C-C motif chemokine receptor 2; HFD, high-fat diet; IL-6, interleukin-6; *Mmp12*, matrix metalloproteinase 12; NF-κB, nuclear factor kappa-B; PVAT, perivascular adipose tissue; Q-VD-OPh, quinoline-Val-Asp-difluorophenoxymethylketone; SMC, smooth muscle cell; *Spp1*, secreted phosphoprotein 1; TLR9, Toll-like receptor 9; TNF-α, tumor necrosis factor-α; VEGF-C, vascular endothelial growth factor C.			

## Gaps in current research

Despite recent advances in elucidating the role of PVAT inflammation in AAA pathogenesis, significant uncertainties and unresolved questions remain. At the preclinical level, most AAA research relies on small mammalian models, particularly mice and rats. A variety of experimental approaches have been employed to induce aneurysm formation, including intraluminal pressurized elastase infusion, chronic angiotensin Ⅱ administration *via* osmotic minipumps, periarterial calcium chloride application, vascular graft implantation, and targeted genetic modifications. Among these, the elastase and Ang Ⅱ models are the most widely used. The elastase infusion model replicates several key pathological features of human AAA, such as leukocyte infiltration, depletion of medial smooth muscle cells, and fragmentation of the elastic lamina. It also effectively reproduces the acute inflammatory phase of aneurysm development. However, thrombus formation and aneurysmal rupture are rarely observed in this model, particularly in mice. In contrast, the Ang Ⅱ model, typically established in hyperlipidemic mice (*e.g.*, *Apoe*^*−/−*^ or *Ldlr*^*−/−*^), is characterized by early macrophage accumulation in the aortic wall^[90]^. Notably, Ang Ⅱ-induced aneurysms predominantly develop in the suprarenal region, whereas human AAA most commonly occurs in the infrarenal segment. The reasons underlying this discrepancy remain incompletely understood but may involve interspecies differences in hemodynamic forces and aortic wall structure^[91–92]^. Given the close relationship between PVAT and systemic adiposity, HFD-induced obesity models are frequently employed to investigate PVAT inflammation. However, HFD alone is generally insufficient to reliably induce aneurysm formation, and the incidence of AAA under such conditions is often suboptimal. Consequently, HFD is commonly combined with elastase or Ang Ⅱ exposure, or with targeted gene knockouts, to elicit a robust aneurysmal phenotype (***Table 3***).

**Table 3:** Experimental evidence relevant to PVAT inflammation in AAA and the models used

Conclusions	Models	References
Neutrophils are attracted by IL-8 and interact directly with adipocytes through the CD11b-ICAM-1 complex.	Indirect evidence from an obesity model	[38]
Neutrophils secrete NE to activate TLR4, thus promoting macrophage recruitment and amplifying inflammation.	Indirect evidence from an obesity model	[39]
EGR1 is differentially expressed between AAA and controls and may be associated with MC activation.	Elastase infusion	[41]
Obesity promotes the release of CCL2 from PVAT and the accumulation of macrophages.	HFD + Ang Ⅱ	[4]
FSP27 facilitates macrophage recruitment *via* the JNK-CCL2 axis and increases MMP-12.	HFD + Ang Ⅱ	[45]
Specific overexpression of SAA in PVAT elevates macrophage infiltration and MMP activity.	HFD + Ang Ⅱ	[48]
Leptin and FABP4 upregulate IL-18, the canonical IL-18 receptor (IL-18R), and NCC, an additional IL-18 receptor identified in AAA lesions, thereby promoting IL-18-mediated inflammatory responses.	Ang Ⅱ + *Apoe^−/−^*	[50]
ATDCs release IL-6, TGF-β, and IL-23, promoting the differentiation of Th17 cells.	Indirect evidence from an obesity model	[51]
Ceramides recruit T cells, and leukocytes likely migrate to aneurysms from the post-capillary venules in PVAT.	Ang Ⅱ + *Apoe^−/−^*	[33]
Overexpression of ET-1 promotes the infiltration of macrophages and secretion of MMP-2.	HFD + *Apoe^−/−^*	[61]
AT1a receptor activation promotes M1 polarization and enhances MMP-2 and MMP-9 through OPN.	Ang Ⅱ + *Apoe^−/−^*	[62]
ANGPTL2 from macrophages could activate the NF-κB cascade and upregulate MMP-9 expression.	CaCl_2_	[63]
Adventitial mast cells can enhance macrophage-derived MMP-9 production through direct contact or IFN-γ signaling.	CaCl_2_	[64]
PDGF-D spurs adventitial fibroblast activation *via* the TGF-β/Smad pathway and exacerbates CD68^+^ macrophage infiltration.	HFD + Ang Ⅱ	[65]
Leptin activates p38 MAPK signaling, downregulating the differentiation markers and upregulating the PCNA in VSMCs.	Indirect evidence from an obesity model	[68]
COMP could significantly increase survivin protein in VSMCs and reduce VSMC apoptosis.	CaCl_2_	[74]
CCL2 mediated the chemoattractant effect of apoptotic VSMCs and triggered macrophage infiltration during SMC death.	Ang Ⅱ + *Apoe^−/−^*	[75]
Abbreviations: ANGPTL2, angiopoietin-like protein 2; AT1a, angiotensin Ⅱ type 1a receptor; ATDC, adipose tissue dendritic cell; CCL2, C-C motif chemokine ligand 2; COMP, cartilage oligomeric matrix protein; EGR1, early growth response 1; ET-1, endothelin-1; FABP4, fatty acid-binding protein 4; FSP27, fat-specific protein 27; ICAM-1, intercellular adhesion molecule-1; JNK, c-Jun N-terminal kinase; MC, mast cell; MMP, matrix metalloproteinase; NCC, Na-Cl co-transporter; NE, neutrophil elastase; NF-κB, nuclear factor kappa-B; PDGF-D, platelet-derived growth factor-D; PCNA, proliferating cell nuclear antigen; TGF-β, transforming growth factor-β; Th17, T helper 17; TLR4, Toll-like receptor 4; VSMC, vascular smooth muscle cell.		

Compared with animal models, human AAA tissue samples present considerable challenges in terms of both quantity and quality, particularly regarding the selection of suitable control tissues. Some studies have utilized aortic tissues from autopsies or surgical biopsies as controls. However, these samples are often affected by RNA degradation, which can compromise transcriptomic analyses and introduce artifactual alterations in gene expression profiles. Moreover, because early-stage aneurysms usually do not meet surgical criteria, obtaining human PVAT samples at this stage is ethically challenging, resulting in a scarcity of related human studies. An alternative approach employed in several studies is to use samples from the non-dilated proximal neck of the same aneurysmal aorta. However, this strategy raises another concern: although this region may appear morphologically intact, it remains uncertain whether it is truly comparable to healthy tissue, given the potential for systemic molecular or genetic alterations in patients with AAA. Taken together, these limitations highlight the persistent challenges in accurately recapitulating the pathophysiology of AAA in current research.

To address these gaps, future research should not only focus on animal models but also strengthen human-based investigation. First, an important direction is to further refine animal AAA models and, when necessary, strategically combine multiple models to strengthen the robustness and translational relevance of experimental evidence. Second, the establishment of standardized human PVAT-aortic tissue biobanks, with unified protocols for sampling, processing, and clinical annotation, could help overcome current limitations associated with small, heterogeneous, end-stage samples. In addition, combining advanced imaging-based assessment of PVAT inflammation with longitudinal cohort studies could provide valuable insights into the early stages of AAA development that are currently inaccessible through surgical sampling. Third, applying single-cell and single-nucleus RNA sequencing together with spatial transcriptomics to human PVAT and adjacent aortic tissues can delineate inflammatory niches, cellular heterogeneity, and interaction networks, thereby providing mechanistic insights that are not attainable through bulk analyses. Finally, systematic multi-omics integration, which combines tissue transcriptomics and spatial data with proteomics, metabolomics, extracellular-vesicle profiling, and paired circulating biomarkers, may facilitate the identification and prioritization of candidate causal mediators that are tissue-localized yet detectable in blood, while supporting the selection of candidate targets for subsequent experimental validation. Collectively, these strategies may provide an actionable roadmap to enhance the validity and translational relevance of future AAA studies.

## Conclusions

The available evidence suggests that PVAT inflammation, as a dynamic and highly plastic process, may contribute importantly to the development and progression of AAA. Disruption of the intricate network of immune cells and signaling factors within PVAT may trigger a cascade of self-amplifying responses that accelerate vascular degeneration. Moreover, the endocrine and paracrine functions of PVAT are closely linked to immune regulation and inflammatory signaling, further amplifying its contribution to disease pathogenesis, and highlighting its potential as a target for AAA assessment and therapeutic intervention (***Fig. 3***). Future studies are needed to validate the effects of PVAT-based pharmacological strategies on AAA.

**Figure 3:**
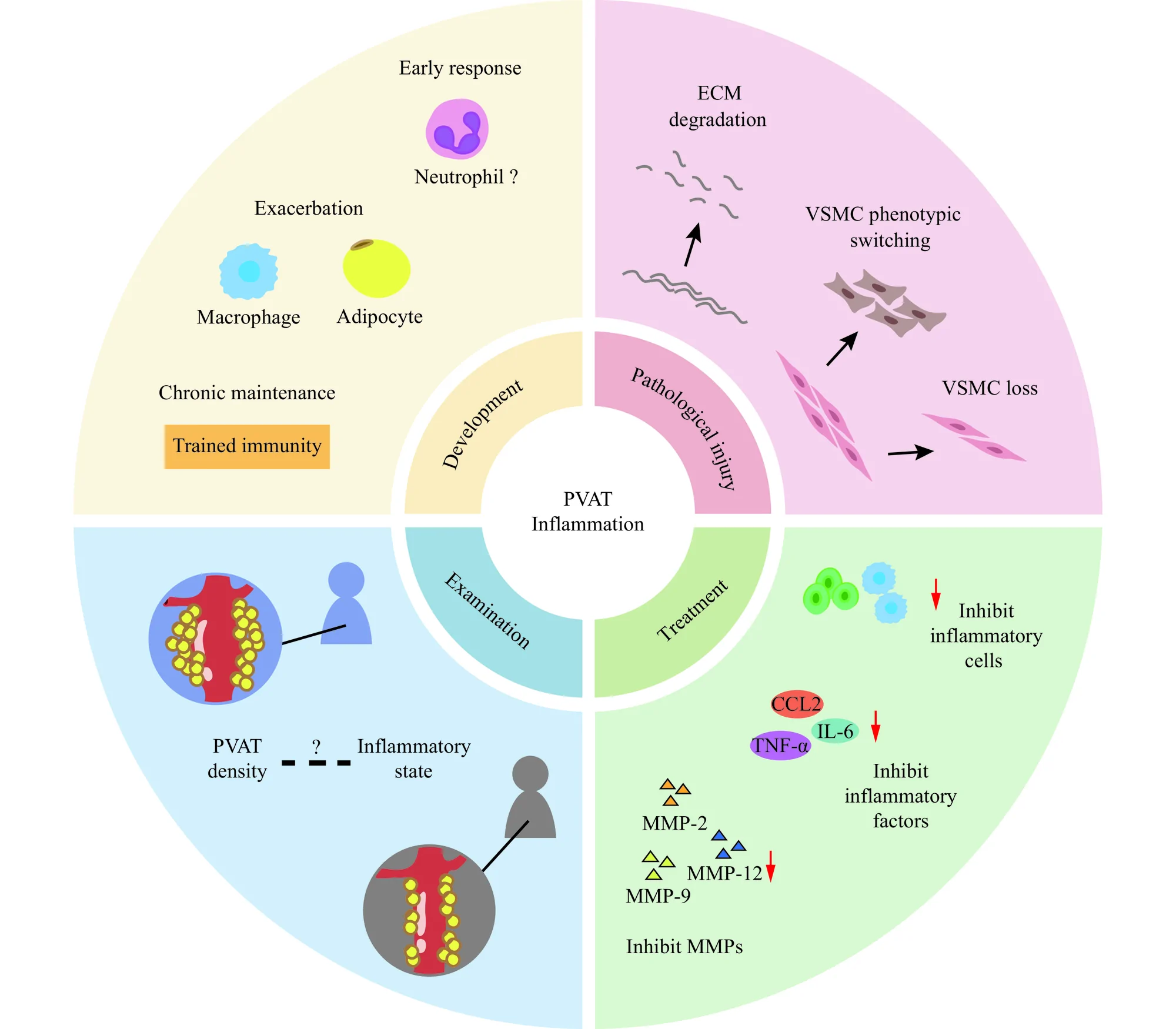
Graphical summary. This figure summarizes the contribution of PVAT-derived inflammation to the progression of abdominal aortic aneurysm, and its potential implications for clinical translation. Abbreviations: CCL2, C-C motif chemokine ligand 2; ECM, extracellular matrix; IL-6, interleukin-6; MMP, matrix metalloproteinase; PVAT, perivascular adipose tissue; TNF-α, tumor necrosis factor-α; VSMC, vascular smooth muscle cell.
